# Performance of artificial intelligence using cone-beam computed tomography for segmentation of oral and maxillofacial structures: A systematic review and meta-analysis

**DOI:** 10.4317/jced.60287

**Published:** 2023-11-01

**Authors:** Farida Abesi, Mahdi Hozuri, Mohammad Zamani

**Affiliations:** 1 Department of Oral and Maxillofacial Radiology, Dental Faculty, Babol University of Medical Sciences, Babol, Iran; 2 Student Research Committee, Babol University of Medical Sciences, Babol, Iran

## Abstract

**Background:**

There are different values reported about the performance of artificial intelligence using cone-beam computed tomography (CBCT) for segmentation of oral and maxillofacial structures. We aimed to perform a systematic review and meta-analysis to provide an overall estimate to resolve the given conflicts.

**Material and Methods:**

A literature search was conducted in Embase, PubMed, and Scopus through 31 October 2022, to identify studies evaluating artificial intelligence systems using oral and maxillofacial CBCT images for automatic segmentation of anatomical landmarks. The surveys had to report the outcome according to dice coefficient (DICE) or dice similarity coefficient (DSC) index. The estimates were presented as percent and 95% confidence interval (CI). I-squared index was used to assess the heterogeneity between the surveys.

**Results:**

A total of 24 eligible studies were finally enrolled. The overall pooled DICE/DSC value for artificial intelligence was 0.92 (95% CI: 0.88-0.95; I-squared=93.6%, *p*<0.001). Tooth and mandible were evaluated more than other anatomical regions (five studies for each one). The lowest and highest DICE/DSC scores for the artificial intelligence related to inferior alveolar nerve (0.55 [95% CI: 0.47-0.63]) and pharyngeal airway and sinonasal cavity (0.98 [95% CI: 0.98-1.00]).

**Conclusions:**

The findings revealed excellent performance for the artificial intelligence regarding the segmentation task of oral and maxillofacial CBCT images.

** Key words:**Artificial intelligence, cone-beam computed tomography, segmentation performance, dentistry.

## Introduction

Cone-beam computed tomography (CBCT) is an x-ray imaging equipment initially used in oral and maxillofacial radiology about two decades ago. This radiographic method can provide high-resolution scans with 360-degree three-dimensional displays (1,2). Compared with traditional CT, CBCT has a shorter scanning time and exposes patients to lower radiation doses (3,4). Despite the popularity of CBCT in dental practice, the accuracy of interpretation of its images can be negatively affected by various factors, such as low interobserver and intraobserver reliability (particularly for junior and less experienced practitioners) (5-7).

Artificial intelligence refers to a wide-ranging branch of computer science that makes smart machines learn and conduct human-like tasks. It can have different systems with complex algorithms potentially providing accurate interpretations by automatic methods (8,9). Dental professionals have benefited from these advantages over recent years and proposed that artificial intelligence could be utilized as a supplementary instrument to enhance the diagnostic performance of other imaging techniques (10,11).

Previous studies tried to integrate CBCT imaging with artificial intelligence applications and investigate their diagnostic performance for the oral and maxillofacial regions to hopefully suggest new artificial intelligence models for clinical practice using CBCT; however, there are variable values reported about the performance of the abovementioned artificial intelligence systems (12,13). Hence, it is necessary to carry out a comprehensive study to resolve the conflicts on this topic. In the current study, we aimed to systematically review the available evidence in the literature on the performance of artificial intelligence using CBCT for the segmentation of oral and maxillofacial structures. For this purpose, we focused on the dice coefficient (DICE) index, which is used to quantify the performance of image segmentation methods. It denotes how much the segmented area is similar to the ground truth (14).

## Material and Methods

-Information sources and search strategy

We searched for the medical literature published through 31 October 2022 in the databases of Embase, PubMed, and Scopus, using the following keywords: *artificial intelligence OR deep learning OR machine learning OR automatic OR automated AND cone-beam computed tomography *OR *CBCT*. The search was limited to the Title/Abstract. We applied no language restriction. We additionally performed hand-searching on the bibliographies of the selected papers. The present study has been reported as per the Preferred Reporting Items for Systematic Reviews and Meta-Analysis (PRISMA) guideline (15).

-Inclusion and exclusion criteria

We enrolled studies that evaluated artificial intelligence systems using oral and maxillofacial CBCT images for automatic segmentation of anatomical landmarks. The articles had to report the outcome according to DICE or dice similarity coefficient (DSC) index. The exclusion criteria were as follows: 1) Reviews, case reports, editorials, and letter to the editors; 2) Duplicate publications; 3) Surveys with unextractable information on the study outcome; 4) Full-texts not being available.

-Study selection and data extraction

The suitability of the identified sources was assessed by independent reviewers through screening the titles and abstracts by use of the pre-designed eligibility forms at the first stage. At the second stage, the full-texts of the potential articles were obtained for more detailed investigations. Any disagreements were resolved by consensus. The following data were extracted for each eligible study: first author’s name, publication year, study country, sample size, artificial intelligence technique, study design, anatomical structure/area, underwent imaging, validation method, DICE or DSC score. We translated non-English reports using the Google Translate.

-Risk of bias assessment

We used the adapted criteria according to the Prediction Model Risk of Bias Assessment Tool (PROBAST) (16) to assess the risk of bias of the included studies. As per the PROBAST, we rate the studies for the risk of bias and concerns about applicability as low, high, or unclear. The details of this assessment tool have been summarized in Supplement 1 (http://www.medicinaoral.com/medoralfree01/aop/jced_60287_s01.pdf).

-Statistical analysis

We pooled the DICE/DSC values of artificial intelligence using a random-effects model. We also estimated the pooled segmentation performance values by the oral and maxillofacial parts as a subgroup analysis. The estimates calculated were presented as percent and 95% confidence interval (CI). I-squared index was used to assess the heterogeneity between the surveys, which ranges from 0.0% to 100.0%; a *p-value* less than 0.10 was considered statistically significant (17). We used forest plots to depict the results of the meta-analysis. Publication bias was appraised using a funnel plot. Meta-regression was used to explore the potential influence of the publication year on the study outcome, and a *p-value* less than 0.05 was considered statistically significant. We performed all statistical analyses by Comprehensive Meta-Analysis V2 software.

## Results

-Search results and study selection

**Figure 1:**
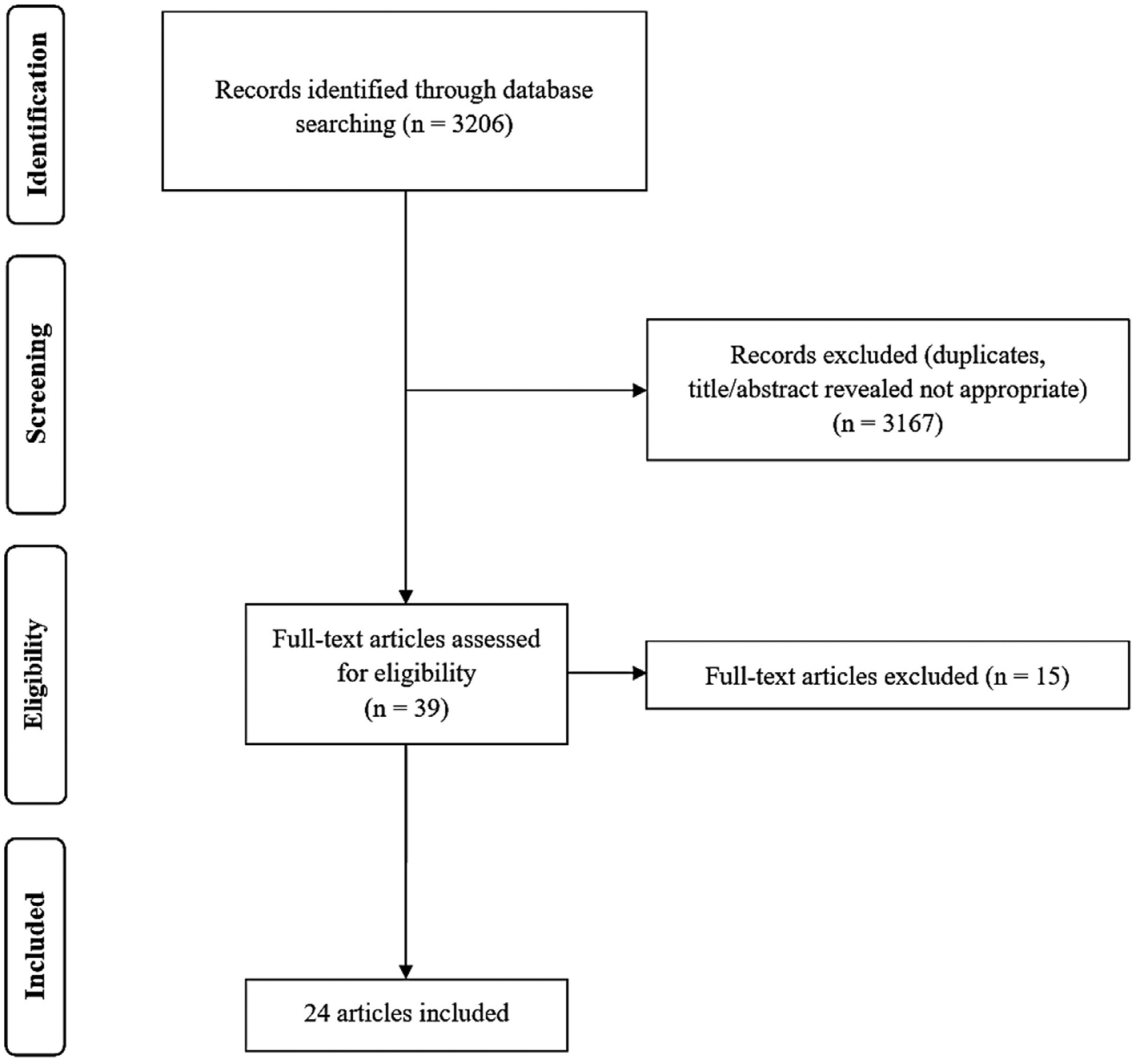
PRISMA flow diagram.

A total of 3,206 publications were initially yielded through the database search. Of these, 39 papers remained after excluding duplicates and unsuiTable sources identified during the title/abstract screening. Full-texts of those potential articles were obtained and assessed. Finally, 24 studies were enrolled in this systematic review after removing ineligible studies (14,18-40). In Fig. 1, a flowchart of the search strategy and results at each step has been illustrated as per the PRISMA.

-Study characteristics

Out of 24 surveys enrolled in this review, there were six studies from Belgium, six studies from China, two from Italy, two from the USA, one from South Korea, one from the Netherlands, one from Turkey, and five multicenter studies. The included studies were reported in English and published between 2013 and 2022. In most studies, deep learning was used as the technique of artificial intelligence (n=21). The baseline information of the included surveys is summarized in Table 1,  Table 1 cont.

**Table 1:**
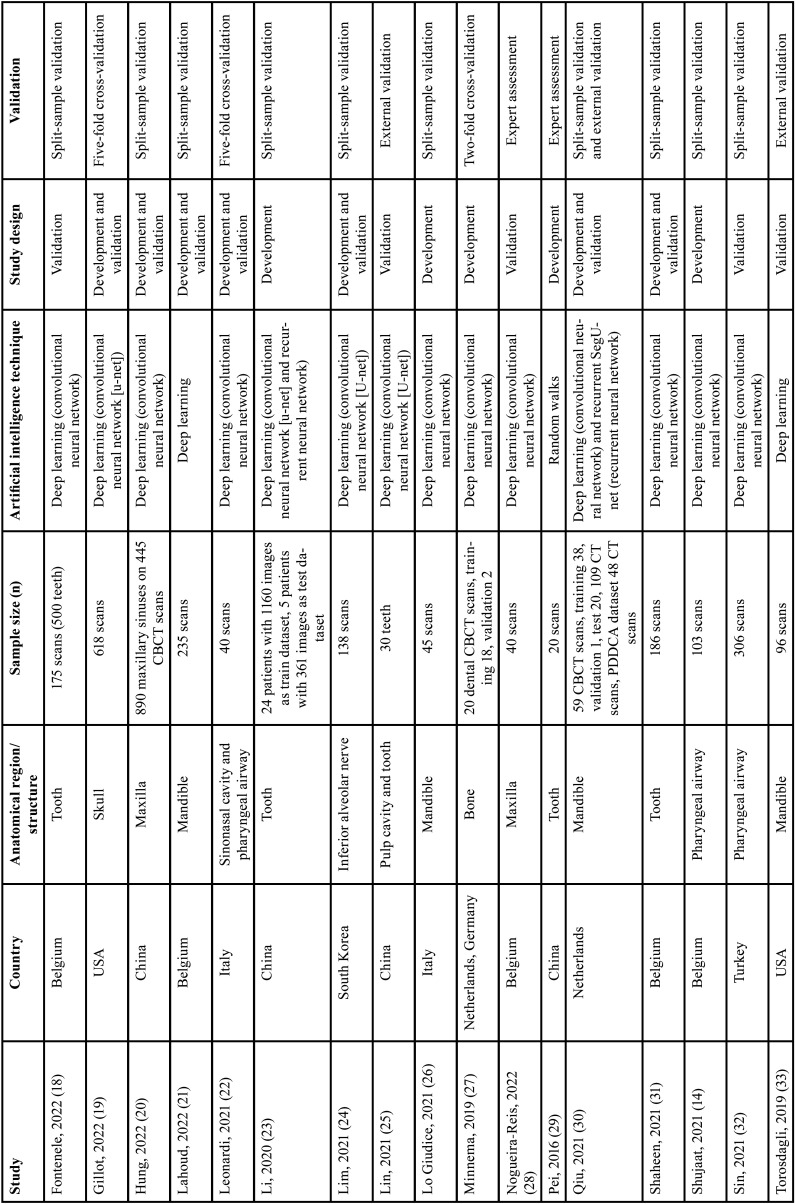
Basic characteristics of studies included in this systematic review.

**Table 1 cont.**
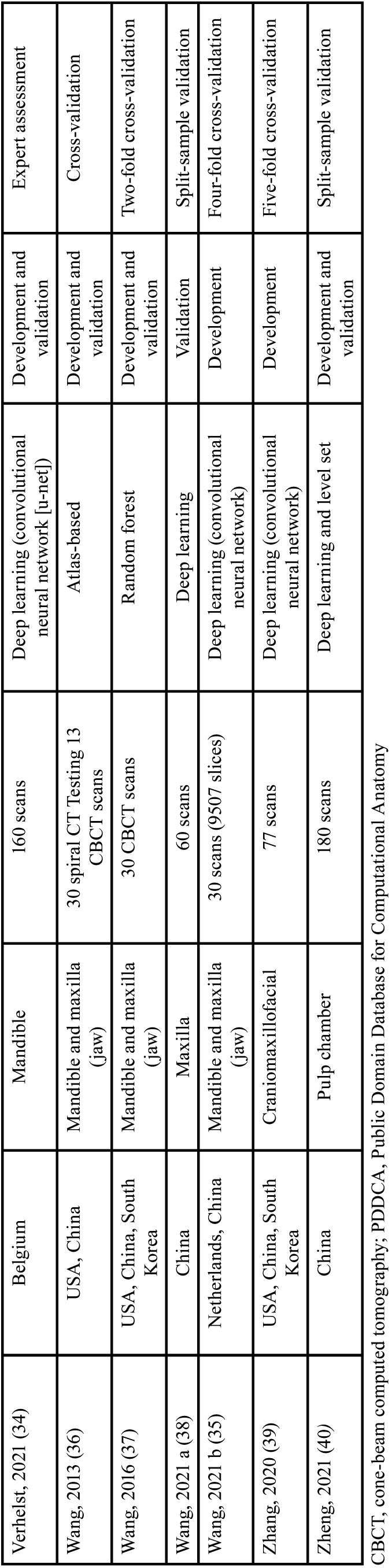
Basic characteristics of studies included in this systematic review.

Meta-analysis findings

**Figure 2:**
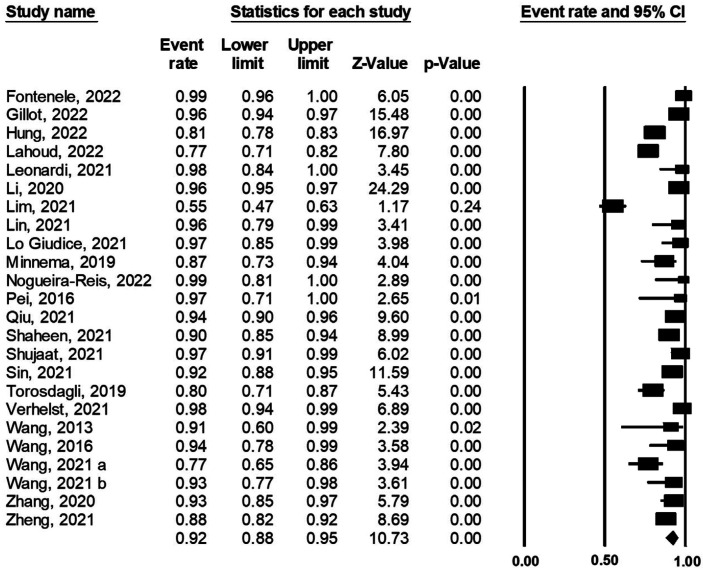
DICE/DSC score of artificial intelligence using oral and maxillofacial cone-beam computed tomography imaging.

**Figure 3:**
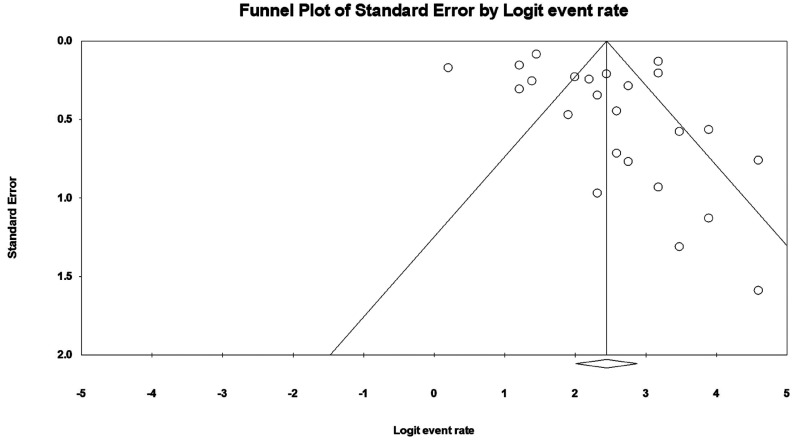
Funnel plot to assess publication bias across studies assessing DICE/DSC score of artificial intelligence using oral and maxillofacial cone-beam computed tomography imaging.

**Figure 4:**
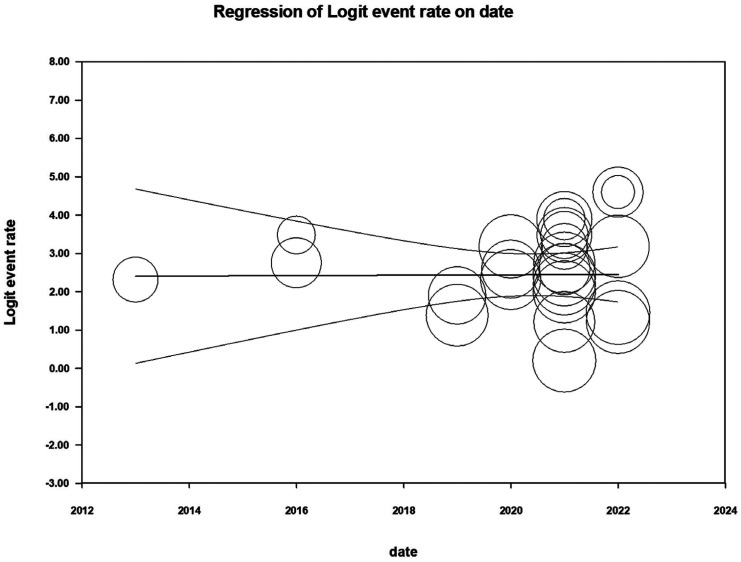
Meta-regression analysis.

There were 24 studies found reporting the DICE/DSC score for artificial intelligence using oral and maxillofacial CBCT imaging. The lowest and highest DICE/DSC index reported were 0.55 and 0.99, respectively. Based on the analysis, the overall pooled DICE/DSC value for artificial intelligence was 0.92 (95% CI: 0.88-0.95; I-squared=93.6%, *p*<0.001) (Fig. 2). The funnel plot was relatively symmetrical (Fig. 3). Meta-regression analysis indicated that publication year did not explain the heterogeneity in the outcome (β=0.005, *p*=0.968) (Fig. 4).

Table 2 summarizes the results of the subgroup analysis by the oral and maxillofacial parts studied. Tooth alone, tooth and pulp cavity, skull, mandible alone, maxilla alone, mandible and maxilla (jaw), pharyngeal airway alone, pharyngeal airway and sinonasal cavity, inferior alveolar nerve, bone, and pulp chamber were the areas assessed. The tooth alone and mandible alone were evaluated more than other anatomical parts (five studies for each). The lowest and highest DICE/DSC scores for the artificial intelligence related to the inferior alveolar nerve (0.55 [95% CI: 0.47-0.63]) and pharyngeal airway and sinonasal cavity (0.98 [95% CI: 0.98-1.00]).

**Table 2:**
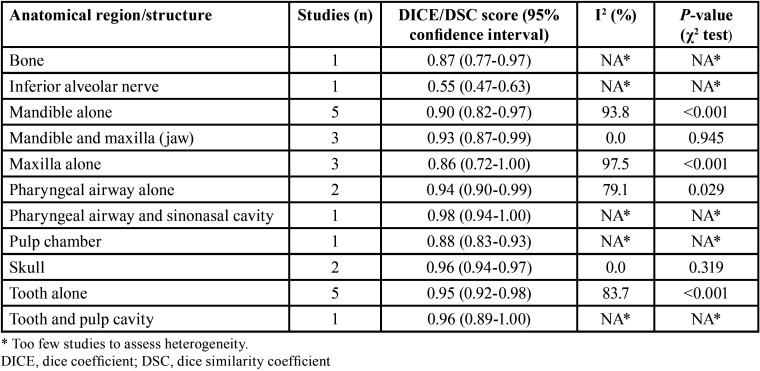
Pooled DICE/DSC score according to anatomical region/structure.

## Discussion

Artificial intelligence systems can now perform different medical tasks even at a higher level of human ability, such as disease diagnosis and treatment; therefore, we witness a significant paradigm shift in the capability of many computer-based tools used in the diagnostic imaging field. Computer-aided diagnosis can also rectify the diagnostic errors happening by humans. However, the validity and reliability of artificial intelligence applications must be clarified before they can be efficiently used in clinical practice. Several studies have endeavored to evaluate the accuracy of artificial intelligence models using oral and maxillofacial CBCT in images segmentation (24,26,31). The segmentation process is actually based on manual or semiautomatic techniques. Therefore, it would be time-consuming and needs expertise. On the other hand, automated computer-based procedures are more effective and clinically appropriate (26). Nevertheless, a comprehensive study has yet to reveal an overall estimate for their performance. Thus, we did a contemporaneous systematic review and meta-analysis of studies reporting the DICE/DSC values for the segmentation of the images by artificial intelligence systems using oral and maxillofacial CBCT.

We searched multiple medical databases and then screened potential citations initially identified using rigorous eligibility criteria. Finally, a total of 24 surveys were included in this systematic review and meta-analysis. Our analysis indicated that the overall pooled DICE/DSC score for artificial intelligence was 0.92, which is an excellent value. However, this rate varied according to the oral and maxillofacial regions; subgroup analysis showed that the lowest and highest DICE/DSC value for the artificial intelligence pertained to the inferior alveolar nerve (score=0.55) and pharyngeal airway and sinonasal cavity (score=0.98).

There are different artificial intelligence techniques for the segmentation of oral and maxillofacial structures, such as deep learning, random walks, atlas-based, and random forest; deep learning has been the most frequently used subset of machine learning. The learning of the deep neural networks is based on extracting characteristics directly from the training data and interpreting the test data (22,23).

Different dental fields are currently benefitting from artificial intelligence systems, such as oral and maxillofacial surgery (diagnosing and classifying structures and guiding surgeons), endodontics (detecting and segmenting the relevant regions), orthodontics (automatic landmark detection and cluster-based segmentation concerning cephalometric analysis), and implantology (qualitative and quantitative appraisal of alveolar bone); however, the segmentation performance of the applications can vary according to the areas studied (12,18,37,40). Based on the present review, the tooth, pulp cavity, skull, mandible, maxilla, pharyngeal airway, sinonasal cavity, inferior alveolar nerve, and pulp chamber were the regions examined in the individual studies included. Artificial intelligence had a weak segmentation performance for the inferior alveolar nerve in the opposite of the pharyngeal airway and sinonasal cavity.

A limitation of the present systematic review and meta-analysis was the high heterogeneity between the included studies, which could be explained by differences in study location, objectives, sample size, scanning device and parameters, presence of noise or artifacts, image acquisition protocols, and interobserver or intraobserver agreement. It should be noted that publication bias could not justify the heterogeneity for the study outcome. Also, the publication year did not explain the heterogeneity in the outcome as per the meta-regression results. Overall, it is suggested to design and carry out more homogeneous investigations.

## Conclusions

The findings of the present systematic review and meta-analysis revealed excellent performance for artificial intelligence regarding the segmentation task of oral and maxillofacial CBCT images. Incorporation of artificial intelligence applications in the oral and dental healthcare systems has the potential to increase the quality of dental care and facilitate the preventive dentistry.
